# Midwives Perceiving and Dealing With Violence Against Women: Is It Mostly About Midwives Actively Protecting Women? A Modified Grounded Theory Study

**DOI:** 10.1177/0886260520927497

**Published:** 2020-06-10

**Authors:** Heidi Siller, Martina König-Bachmann, Susanne Perkhofer, Margarethe Hochleitner

**Affiliations:** 1 Medical University of Innsbruck, Austria; 2 Health University of Applied Sciences Tyrol, Innsbruck, Austria

**Keywords:** disclosure of domestic violence, domestic violence, perceptions of domestic violence

## Abstract

Violence against women (VAW) affects pregnancy, birthing, and puerperium. In this sense, domestic violence (DV) or intimate partner violence (IPV) may increase during pregnancy, sexual child abuse may affect the birthing process, and obstetric violence may occur during birthing. Thus, consideration of violence during pregnancy and puerperium is essential to providing optimal care for women. This implies that midwives should be able to identify women affected by VAW. Therefore, this study explored VAW as perceived by midwives in one region of Austria. Interviews with 15 midwives (mean age 38.7 years) were conducted in Tyrol, Austria, between December 2014 and December 2015. Data were analyzed with a modified version of Grounded Theory. The core category “protecting but walking on eggshells” showed the caution with which midwives approached VAW and in particular DV or IPV. Including VAW in midwifery was connected to midwives being active protectors of women in their care. This meant being an intuitive, sensible, guiding, and empowering midwife to the woman. Staying active was necessary to fulfill the protective role also with regard to DV. However, this was influenced by the visibility of the connection between VAW, pregnancy, childbirth, and puerperium. The key to including VAW and particularly DV was midwives’ professional role of actively protecting women. Neglecting DV during pregnancy, labor, and puerperium meant further silencing DV, and keeping it hidden. Considering these aspects in planning and implementing actions to prevent VAW is expected to support sustainability and motivation to ask women about all forms of violence during and after pregnancy.

Any attempt to research violence against women (VAW) over the life course means focusing on periods of potential vulnerability. For example, during pregnancy, childbirth, and puerperium, women may be particularly vulnerable to VAW or particularly impacted by having experienced violence in the past. Violence in this context refers most often to childhood abuse, domestic violence (DV), intimate partner violence (IPV), and obstetric violence (OV). OV, also known as disrespect, abuse, or mistreatment during childbirth, is mostly perpetrated by medical and health care staff (Jardim & Modena, 2018).

This article focuses on midwives’ perception of VAW in Austria. Studying this subject is crucial to facilitate the implementation of training regarding responses to VAW. A qualitative study with six midwives in Jamaica showed that midwives without formal training about VAW perceived their professional role to include intervening when caring for a woman experiencing gender-based violence (Pitter, 2016). Another qualitative study with 21 midwives in Australia found that routine inquiry about IPV was seen as an integral part of midwifery; simultaneously, midwives reported lack of training and lack of support to fully embrace it (Eustace et al., 2016). Similarly, an Italian qualitative study with 15 midwives found that midwives believed they had a critical role in the detection and response to violence; however, they reported having insufficient training and uncertainty regarding the detection of DV (Mauri et al., 2015). In Great Britain, focus group findings of 11 midwives who received training on DV found that they took pride in supporting women who had experienced DV and perceived asking about DV as a critical part of their professional role (Baird et al., 2013). Despite these findings, research on midwives’ perception has been relatively limited, particularly regarding VAW in its entirety. Such a lack of research is particularly true for Austria. Therefore, we aimed to further close this gap in the literature.

In 2014, the European Union Agency for Fundamental Rights (FRA, 2014) published the largest survey to date on VAW in the European Union, comprising 42,002 participants. According to the survey results, in Austria, 31% of 1,505 women reported physical, sexual, and/or psychological childhood abuse compared to 35% in the European Union (FRA, 2014). Thirteen percent reported sexual and/or physical violence by a partner since the age of 15 (FRA, 2012). We are unaware of any current data on IPV during pregnancy in Austria. However, in the European Union, 20% of 1,762 women who had experienced violence in a relationship had also experienced violence during pregnancy by their current partner, while 42% out of 3,120 women had experienced violence by a previous partner (FRA, 2014).

Internationally, studies have demonstrated a decline or increase in DV or IPV during pregnancy or puerperium. For example, in a Turkish survey, 47.6% of 317 women reported DV over their life course, which decreased to 10.3% during pregnancy (Bagcioglu et al., 2014). Moreover, a survey of 82 women in Japan reported a decline from 34.9% to 20.7% in IPV during pregnancy (Kataoka et al., 2016). In a Canadian survey, 6% out of 76,500 women reported physical and/or sexual abuse before pregnancy, while 2.5% had experienced some type of abuse before, during, or after pregnancy. However, of those mothers who experienced abuse, approximately half reported its onset during pregnancy (Daoud et al., 2012). Similar findings were presented in a Swedish longitudinal survey of women during pregnancy and up to one to one and a half years postpartum. Out of 720 women, 2.5% had experienced abuse solely during pregnancy, whereas DV increased to 3.3% in the one to one and a half years postpartum (Finnbogadóttir & Dykes, 2016).

The influence of IPV and DV during pregnancy is widespread and affects the health and quality of life of both mother and infant. Adverse health effects resulting from IPV, such as depression, anxiety, and suicidal behavior, might be amplified in pregnancy (Chisholm et al., 2017; Howell et al., 2017). Multiple reviews of empirical studies have demonstrated that IPV before pregnancy (Nesari et al., 2018), IPV during pregnancy (Alhusen et al., 2015; Hill et al., 2016), or the experience of childhood abuse (Hutchens et al., 2017) are connected with an increased risk of postpartum depression, preterm delivery, or the infants’ low birth weight. Other systematic reviews and meta-analyses illustrated that the experience of IPV also reduced the likelihood of receiving adequate antenatal care (Musa et al., 2019), delay in accessing antenatal care (Jamieson, 2018) and increased health risks for the woman and neonate, such as poor nutrition, inadequate weight gain, increased rates of smoking, and substance or alcohol abuse (Alhusen et al., 2015). DV or IPV thus affects the neonate through an increased risk of spontaneous abortion, preterm labor, or low birth weight, as evidenced by a literature review (Alhusen et al., 2015) and a survey of 468 victimized women in Australia (Meuleners et al., 2011).

The birthing experience is also affected by violence. Pregnant women who have experienced childhood sexual abuse (CSA) might be reminded of aspects of the abuse during pregnancy examinations or when giving birth. Birth was found to serve as a trigger of memories of sexual abuse in several studies: a German survey of 85 women with CSA experience compared to 170 control women (Leeners et al., 2016), a qualitative study with eight women in the United States who had experienced sexual abuse (LoGiudice & Beck, 2016), and in a further study with nine women in Great Britain with CSA experience (Montgomery et al., 2015). Therefore, health care staff must be aware of the impact of violence on both health and the birthing experience. Another important consideration is that the birthing process itself can be a source of violence toward women; such violence is described as OV (Jardim & Modena, 2018). A European study has demonstrated that, out of 6,923 women, 20.7% reported abuse in health care over their lifetime, and the experience of such abuse and the severe suffering it caused were associated with fear of childbirth (Lukasse et al., 2015). Such abuse led to a global campaign to raise awareness of OV, known as the Roses Revolution (2013), which advocates human rights during childbirth. Women who had experienced OV during childbirth were encouraged to put roses in front of the delivery room to draw attention to this matter and stimulate change.

Routine inquiry is crucial to address VAW; women affected by VAW need to be identified and referred to specialized services. Routine inquiry is known to increase the identification of victimized patients, as evidenced by a systematic review by O’Reilly et al. (2010). Midwives play an essential role in the care of women during pregnancy, childbirth, and puerperium. However, qualitative studies with 21 midwives in Australia (Eustace et al., 2016) and 15 midwives in Italy (Mauri et al., 2015) demonstrated that midwives feel inadequately prepared to ask about violence. Qualitative studies of, for example, 21 midwives in Australia (Eustace et al., 2016) and 6 midwives in Jamaica (Pitter, 2016) demonstrated that midwives worried about resources for those affected by violence, while eight midwives in Norway wished for support structures for midwives (Henriksen et al., 2017).

Health services should also be guided by a woman-centered approach to increase the self-efficacy of victimized women as well as a respectful and positive childbirth experience (World Health Organization [WHO], 2017). Such woman-centeredness is promoted in contemporary midwifery (Berg et al., 2012; Bradfield et al., 2018; Bradfield, Hauck, Kelly, & Duggan, 2019). Woman-centered care includes a “with-woman” approach. In an Australian qualitative study with 31 midwives, midwives emphasized the importance of being with the woman in contrast to doing midwifery “to” the woman (Bradfield, Hauck, Duggan, & Kelly, 2019). The with-woman approach is essential to the midwife’s professional identity and includes the partnership between midwife and woman, and woman-centered practice, such as informed decision making (Bradfield, Hauck, Duggan, & Kelly, 2019). Woman-centered care focuses on the importance of the relationship between woman and midwife, collaboration, trust, reciprocity, and respect, as shown in a qualitative study of 10 midwives in Australia (Bradfield, Hauck, Kelly, & Duggan, 2019) and a review by Fontein-Kuipers et al. (2018). Midwives attempt to protect and guide the women in their care as highlighted in a grounded theory study based on 29 interviews with midwives, observed and tape-recorded booking interviews between midwives and pregnant women in the United Kingdom (Levy, 2006). As also shown by Fontein-Kuipers et al. (2018) and in a survey with 280 midwives and midwifery students in Switzerland (Oelhafen & Cignacco, 2018), midwives consider women’s needs and wishes. In their qualitative study with 10 midwives in Sweden, Thelin et al. (2014) described midwives as being “guided guide[s]” (p. 115). For example, as active professionals, midwives provide information about pregnancy, childbirth, and puerperium, guide women in their care, provide a basis for collaboration, and actively protect women. Such an understanding of their profession might integrate VAW as one topic of care in midwifery.

In Austria, midwives may be based in a hospital or private practice, and they care for women during pregnancy, labor, puerperium, and the neonate’s first year. The compulsory health insurance system in Austria (Federal Ministry of Labour, Social Affairs, Health and Consumer Protection, 2019) covers midwife visits before and after giving birth. In Austria, training in midwifery does not yet systematically include the subject VAW (Erdemgil-Brandstätter, 2016). The lack of systematic training may also be connected to a lack of obligation to routinely enquire about VAW in the context of pregnancy, despite WHO (2016) recommendations to do so.

Against this backdrop, we considered it necessary to study midwives’ perceptions of VAW in Austria. We focus on VAW to examine VAW in its entirety, but we refer to specific forms of VAW such as DV, IPV, or OV if required by the context. To understand how midwives respond to VAW when caring for women, we used a modified version of the grounded theory approach. This study adds to the extant literature by focusing on midwives’ perception of VAW in Austria.

## Materials and Methods

### Participants

We used a convenience sample for this study (Robinson, 2014). Eligible participants included midwives in Tyrol, Austria. In this part of Austria, there are currently no men working as midwives. Therefore, the study consisted solely of female participants. Midwives were invited to participate in the study at the annual regional plenary meeting of midwives organized by the regional midwives’ association. In addition to recruiting participants on-site, all midwives in this part of Austria were sent an email about the study. This was done to ensure that those who had not attended the meeting were also reached and to increase the sample diversity. Two email reminders were sent during the recruitment process as well as spreading information by word-of-mouth. Participation was voluntary.

Diversity in participants was achieved for age, years of work experience as a midwife, and working environment (clinic/private practice). Participants had a mean age of 38.7 (range 24–53) years and had been practicing as midwives for an average of 17.2 (range 2–32) years. At the time of the interview, most (*n =* 11) participants were working in private practice, while three were working in a clinic, and one was currently employed in both settings. Six midwives reported having had additional training that also focused on some form of VAW. Information on age, years of work experience, and training on VAW or DV is shown in Table 1.

**Table 1.** Participant Characteristics.

Midwife (Interview Number)	Age (Years)	Years of Experience as a Midwife (Range in Years)	Training in VAW or DV (Yes/No)
1	52	30–35	No
2	47	25–30	Yes
3	53	25–30	No
4	35	Missing	Yes
5	26	Missing	No
6	44	20–25	Yes
7	27	5–10	No
8	39	1–5	No
9	24	1–5	Yes
10	51	30–35	No
11	24	1–5	No
12	37	10–15	No
13	50	25–30	Yes
14	42	20–25	Yes
15	29	5–10	No

*Note.* VAW = violence against women; DV = domestic violence.

### Ethics

The study was approved by the Research Committee of the Health University of Applied Sciences Tyrol and the Ethics Committee of the Medical University of Innsbruck, Austria. All participants provided written consent for participation and the anonymous use of their data. All personal information in the transcripts (e.g., names, working site) was anonymized to ensure that it could not be traced back to the individual participant.

### Conduct of Interviews

Of 66 potential candidates who attended the annual meeting, 15 midwives participated in individual interviews between December 2014 and December 2015. The time and place for interviews were agreed upon by the midwife and interviewer (HS). Participants selected the interview location: 12 interviews were conducted at the participants’ workplaces, two at the participants’ homes, and one in the interviewer’s office. All interviews were conducted in a separate and interference-free room. Interviews were conducted face-to-face with only the interviewer and interviewee present and had a mean duration of 42 (range 24–67) minutes. The interviewer was a psychologist with experience in conducting interviews and analysis. All interviews were audio-recorded and transcribed verbatim.

### Interview Guideline

Problem-centered interviews (Witzel, 2000) were considered to be the most suitable for this study, to examine how midwives perceived, discussed, and dealt with violence in their care of women. According to Witzel (2000), problem-centered interviews are conceptually built on procedures to generate theory (cf., grounded theory) and follow a cyclic inductive and deductive procedure by gradually collecting and verifying data. A position of general openness to empirical observation informed the interviews, but allowed knowledge on the subject to be introduced. This knowledge comprises a framework for ideas and a basis for dialog between interviewer and interviewee (Witzel, 2000), but the interviewee determines the relevance of these topics in their narration. In addition, interviews are guided by a problem-centered orientation toward socially relevant issues, such as VAW. In this sense, interviewees are conceived experts regarding their own actions (Witzel, 2000) and thus, in the context of this study, with regard to how VAW is discussed in midwifery and their perception of VAW in midwifery. An interview guideline was developed from literature research regarding how VAW is addressed in health care and midwifery. This guideline was used as a prompt but allowed ad hoc questions to be asked if certain topics were not mentioned. This procedure ensured comparability between interviewees (Witzel, 2000) and the ability to adapt the interview guideline as required.

We used opening questions (Witzel, 2000) regarding the midwife as a person and her professional background, as well as about a typical day as a midwife (First, I would like to ask you about yourself and your work as a midwife. Could you please tell me about your everyday life as a midwife?). Thereafter, questions were asked to specifically explore VAW in midwifery. Thus, midwives were asked about their experiences with women affected by VAW (e.g., What kind of experience do you have with patients who have experienced violence?; How do you perceive violence in pregnant women?) and regarding their self-care when dealing with sensitive topics (e.g., How do you address the knowledge that your patients have experienced violence?). Midwives were asked for their opinions, expectations, and concerns regarding routine inquiry for violence in midwifery (e.g., What do you think about routine inquiry regarding violence in pregnant women?).

### Data Analysis

HS and MKB engaged in data analysis using a modified version of grounded theory based on Strauss and Corbin’s (1990) method. As recommended by Corbin and Strauss (1990), data analysis and interviews were conducted alternately to ensure a cyclic process of data acquisition and analysis (Strauss and Corbin, 1990; Strübing et al., 2018), which meant that we analyzed the first five interviews and drafted concepts before returning to data collection. Eventually, we felt that sufficient conceptual depth and saturation had been reached, as a certain range of data had been established in each category (Nelson, 2017). Strübing et al. (2018) postulated that empirical saturation does not depend on the number of participants or interviews, but that saturation can be observed in the range within data (e.g., deviant cases, breadth of phenomenon); intensity of analysis (the cyclical nature of data acquisition and analysis); and the interaction between field and researcher. This understanding of empirical saturation overlaps with definitions of theoretical saturation, which focus on the variation of properties in concepts and categories (Charmaz, 2006; Low, 2019; Saunders et al., 2018). Saturation in our data was partly demonstrated in the frequency of text assigned to a category, and more particularly, in the diversity and complexity of a given category. This is also in line with theoretical saturation, which promotes searching for variation in concepts and categories (Guest et al., 2006). For example, when analyzing the first interviews, we noticed that the picture of how midwives responded to DV was incomplete, and the categories lacked variance regarding the perspectives on addressing VAW. After continuing with sampling and analysis of additional interviews, variations in the categories expanded and enriched the understanding of dealing with VAW in midwifery. Recruiting participants also meant focusing on midwives working in rural and urban areas to include a variety of perspectives. After analyzing 15 interviews, we felt that we had captured the full extent of the phenomenon of VAW in midwifery in our regional context. When comparing our findings with the literature, we observed a resonance (Nelson, 2017), although our findings also had novel aspects that were not as explicit in other research on VAW and midwives.

The analytical procedure can be summarized as follows. The first phase of analysis included opening up the data by asking questions regarding the actors, the inclusion of VAW in midwifery, perception of violence, experiences with those affected by VAW, and its relevance to midwifery. After line-by-line coding, all coded sequences were collected, compared, and categorized to create concepts (Mey & Mruck, 2011; Strauss & Corbin, 1990). These steps of open coding, sorting codes, and categorizing codes into concepts were conducted using the software Maxqda^®^ (2018), whereas further analysis was conducted manually by, for example, paper-and-pencil drawing of pathways and possibilities regarding how VAW is perceived in midwifery and writing up storylines. After the first steps of coding, HS and MKB compared their coding and concepts and discussed inconsistencies. To reach a consensus for any inconsistencies, the initial steps of open coding and sorting codes into concepts were repeated together, alongside discussing initial ideas and aspects of the coding. This procedure clarified inconsistencies. The ideas regarding data were written down as memos and included throughout the next phases of the analysis process, more particularly when developing the storyline and drafting the theory.

The second phase of analysis was intended to develop categories based on the concepts established in the beginning. Throughout the analysis, codes were compared to codes and concepts, and concepts to concepts and categories, and categories with each other. This constant comparison grouped concepts into categories, but also allowed the expansion of properties within each category. In addition, a focus was placed on interactions and relations between categories during axial and selective coding. Context, interaction, and relation to/between categories were obtained using the paradigm model (Strauss & Corbin, 1990), which also supported the development of the central phenomenon (also referred to as the core category). The paradigm model focuses on the central phenomenon in the data and the condition that brings the phenomenon to the fore. It also includes the context in which the phenomenon occurs, and what actions and interactions it generates, fosters, or hinders. Thereby, it is also important to examine any intervening aspects that might bring in change. Finally, the paradigm model provides information on the consequences that result from these dynamics (Strauss & Corbin, 1990).

The third phase of analysis included the development of the model and overlapped with selective coding, thereby following a cyclical process of analysis. HS and MKB wrote storylines to grasp the essential aspects and further elaborate the core category in the data, to clarify the interaction and intersection of categories (Strauss & Corbin, 1990), and to construct a theory based on the data (Birks et al., 2009). This theory is understood to explain the phenomenon and provide a framework (Rodner, 2019) in which VAW in midwifery can be comprehended. The findings were presented at the subsequent regional midwifery association’s plenary meeting of midwives to allow attendees to provide feedback regarding whether their reality had been accurately represented (Thomas, 2017).

### Reflexivity

The professional context produces frames of meaning for analysis and interpretation of data/findings (Bergman & Coxon, 2005; Rodner, 2019). Reflecting on this is a criterion for the quality control of data (O’Brien et al., 2014; Strübing et al., 2018). As a member of the midwifery community, MKB had a close connection to the field that allowed access to the field, facilitated recruitment of participants, and provided insight into the development of the midwifery profession and its current status. However, it also meant actively distancing herself from implicit knowledge. This included conscious reflection and self-awareness during data analysis. HS is a psychologist not connected to the field of midwifery. This facilitated the conduct of interviews, as there was no relationship to the participants and no implicit knowledge of midwifery.

## Results

The categories connected to the core category “protecting but walking on eggshells” illustrated how midwives cautiously circled the topic of VAW. The term “walking on eggshells” was selected to demonstrate the caution and skepticism with which participants broached the topic. The skepticism in midwives was noticeable because they doubted that pregnancy would be the right time to ask about VAW, were unsure how to ask about VAW, or unsure about a smooth referral to specialized services. Simultaneously, being a midwife meant protecting women, empowering them in decision-making processes, and increasing their self-determination and control during examinations or childbirth. DV and IPV stood out and had a specific status compared to other forms of VAW. This meant that DV and IPV were dealt with differently and fostered insecurity in midwives. Thus, midwife’s insecurity connected to DV and IPV did not appear in other forms of VAW. Their insecurity related to how to ask about DV and IPV, and what steps to take after the disclosure of DV and IPV. The category *different shades of violence* demonstrated that DV included IPV and sexual abuse in childhood, whereas VAW was also observed in OV and distressing experiences such as fetal death, child removal, or substance abuse. Most forms of VAW (except DV) were seen as relevant to midwifery, particularly as these were visibly linked to childbirth and puerperium. The link between DV and childbirth appeared to be relatively hidden, which resulted in midwives paying little attention to DV in their dealings with women, compared to other forms of VAW. For example, OV affects the woman immediately when the midwife is present. The same visible connection can be observed with regard to fetal death and stillborn neonates, or child removal. This visible connection calls the midwife to action due to her professional role as guide and protector. However, DV most often does not occur during labor or in the presence of the midwife, which makes the connection more hidden. Midwives reported suspecting DV from women’s behavior during childbirth (e.g., being very tense and heightened fear of childbirth), which resulted in behavior adjustment on the part of the midwife, by, for example, reducing the number of vaginal examinations and increasing a sense of control in women during examinations. The concealed connection between DV and labor contributed to midwives silencing and neglecting direct and active questioning about DV. Instead, midwives’ misconceptions that DV is a private matter and a taboo subject led to more silencing, increased insecurity regarding how to broach the subject, and heightened caution about addressing it; thus, they approached the subject as if they were walking on eggshells.

Despite the relationship with DV in terms of hesitation and insecurity to discuss DV, midwives also voiced their intentions to broach the topic of VAW, as illustrated in the category *breaking barriers*. Ambivalence toward DV arose in this context because participants faced barriers, such as lack of training or skills, as well as personal hesitation in discussing DV with women in their care. Despite viewing screening questions about DV as the midwife’s domain, and perceiving VAW generally as an important topic, the ambivalence toward these topics increased when midwives were concerned that they might be failing women. Without training on VAW, midwives doubted whether an interlocking referral system was available and also discussed limitations in their scope of action. In this sense, midwives were encouraged to collaborate with other professionals. In some settings (i.e., hospitals), this was easier due to the interdisciplinary setting. This was also evident when highlighting the importance of interdisciplinary networks in the DV context. Simultaneously, midwives also reported a lack of knowledge regarding ways to address DV. It was notable that ambivalence and hesitation to address DV were overcome if participants had received some training on DV or VAW, and thereby the connection between pregnancy, childbirth, puerperium, DV, and VAW became visible and prepared them for the actual question and positive disclosure. This included being prepared before asking the questions, knowing what to do next, as well as personal preparedness, such as knowing how to respond to positive disclosure on an emotional level.

**Figure 1.**
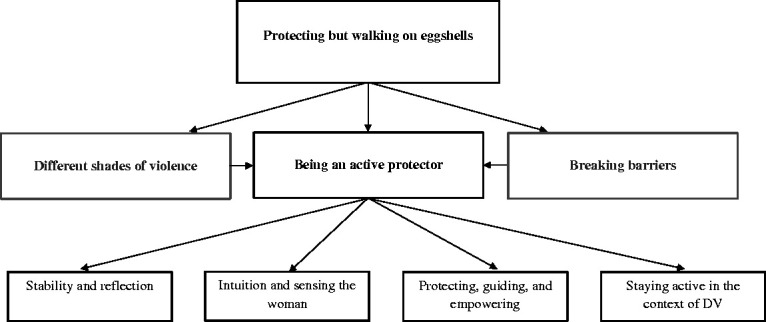
Connectedness between core category and categories. The core category (protecting but walking on eggshells) illustrates the midwives’ cautious circling of the topic of VAW, while also protecting and empowering women in their care. Related categories referred to midwives’ discussion of various forms of VAW (different shades of violence); midwives’ professional role as active protectors of women (being an active protector); and their intention to broach the topic of VAW (breaking barriers). The focus of this article is on the category “being an active protector,” which connects to four themes.

Most importantly, discussing VAW in its entirety was not only dependent on having received training but was influenced by the professional role of midwives as represented in the category *being an active protector* (see Figure 1). In general, the ability to distance oneself from challenging situations, having coping strategies that reduced stress, and a trustful relationship were important assets in midwifery. Sensing the woman was characteristic for midwives and reinforced protecting women from harm and supporting women by guiding them, which promoted the empowerment of women and increased women’s self-efficacy and self-determination during pregnancy, labor, and puerperium. This meant that, being a midwife was also about being a protector and guide for women during pregnancy, childbirth, and puerperium. To protect and guide, midwives intervened in visible forms of VAW, such as OV. Strengthening the midwife’s role as protector and guide for victimized women meant uncovering the hidden connection between DV and pregnancy, childbirth, and the postnatal period.

The following section focuses on the description of the core category and the category of being an active protector. It illustrates an approach for actively including DV in midwifery and highlights the possibilities of including this approach in the professional role of midwives and explaining the associated difficulties.

### The Core Category “Protecting but Walking on Eggshells”

The core category represented the overall phenomenon that connected all categories. The core category illustrated that the midwife’s professional role is anchored in actively protecting women, despite midwives being cautious about addressing VAW. Protection referred to shielding women in case of violations or emotional transgressions during the birthing process and also talking to women and empowering them in case of challenging situations such as stillbirth. Such protection was guided by visible transgressions and violations and was less noticeable in the context of DV. Walking on eggshells indicated that midwives tread very sensitively and carefully on the topic of DV and were skeptical regarding its inclusion in midwifery. Such skepticism was apparent in their questioning whether the pregnancy was a suitable time for routine inquiry about DV. The following statement also illustrates the general positive attitude toward addressing DV, but a simultaneous questioning of its suitability in pregnancy.

In principle I think, I always think that a routine inquiry [regarding DV] is something good. But there is again this question, is it sensible to do something like that during pregnancy? (Midwife 9)

Another midwife doubted if routine inquiry worked in everyday midwifery because “in our daily work there is neither rhyme nor reason in that in the end” (Midwife 7). In this context, there was also reference to a missing systematic strategy to include routine inquiry in midwifery. Participants endorsed routine inquiry but simultaneously referred to difficulties in realization, such as “when and in which context and who does [routine inquiry] is difficult” (Midwife 8); or difficulties posing the question because “I don’t know how to ask, to be honest, I cannot imagine how to ask about it best” (Midwife 10). Such a lack of training, skills, and knowledge regarding how women would react was noticeable throughout the interviews. Caution about posing the question also touched on midwives’ own competence, authenticity, and well-being when asking about VAW. In the example below, a midwife who had some training in DV highlighted the need to trust one’s skills and knowledge and to be prepared for what to expect.

I can trust myself to be able to deal with this situation, which means, if a woman tells me about her experiences with violence, I must have the courage to enquire. I think you should not be afraid to be honest in such a situation. And do not sugar-coat or appease it, you can also allow things to stand as they are, but you should not be afraid. (Midwife 4)

Despite the caution concerning VAW in midwifery, the care of women was characterized by protecting and guiding them. One midwife described that protection against OV was particularly necessary, thereby also touching on the visible link between VAW and childbirth experiences.

As a midwife, you have a great responsibility. Precisely because sometimes you feel that you have to protect the women from insensitive health professionals. (Midwife 8)

Such protection also meant providing a safe space for the woman, because “primarily you want to increase the sense of safety of the woman” (Midwife 2). This sense of safety also referred to women with a history of DV. Protecting did not stop at the active protection of women, but also encompassed encouraging and empowering them. Thereby, midwives were able to touch on the subject of DV. Even when not addressing it directly, they tried to prevent the women from being further harmed by increasing their sensitivity in their actions.

### Being an Active Protector

The following illustration of the category *being an active protector* commenced with the premise that the professional role of midwives was associated with descriptions of strong, sensitive, independently acting, and empowering protectors of women. Being an active protector also meant initiating actions when guiding and protecting women. Being an active protector was seen in four themes, which are discussed below: (a) the effect of violence on midwives’ emotional level (stability and reflection); (b) interaction between midwife and woman (intuition and sensing the woman); (c) increasing the woman’s self-efficacy (protecting, guiding, and empowering); (d) the midwife in relation to other professionals and her sense of involvement in the context of DV (staying active in the context of DV).

#### Stability and reflection

The professional role meant the midwife reflecting on her own history and its influence on the interaction with women in her care. In this way, the midwife focused on herself and her emotional balance. One midwife described the importance of knowing one’s own triggers to cope with stress and challenges:

That is a very important topic, namely that you also include your own stories and that you ask yourself “what am I going to do with [this experience]?” or “why do I react in a certain way in these specific situations?” or “what happens with this word, that suddenly my pulse is accelerating” or something similar. (Midwife 8)

Such reflection also referred to the midwife’s own history of violent experiences and how it might affect them caring for victimized patients. Being a midwife meant providing the best possible support for the woman and adapting one’s support to whatever is required:

Before you get to know the woman, you get her case file. And when reading it you already tear up, and then the woman is sitting in front of you, in blood and flesh, and it is hard, really hard, and of course, it affects me. Then I think I can only reach out to her, to wherever she stands right now. I can only be here for her in that very moment. [.] how shall I be able to change her past [.] I embrace her the way she is now, I take her story, and I work with her in the here and now. (Midwife 4)

This approach demonstrates how midwives use their skills of reflection in distressing situations, but also how they use this reflection to respond to the emotional effect of these encounters and focus on the woman’s needs in the given situation. Reflection and stability in midwifery also meant protecting oneself from stress and emotional overload, being grounded in one’s actions, drawing a professional line between stress at work and leisure/family time, and having one’s own peer network. Midwives coped with distressing encounters by talking to other midwives in their team to balance their emotional needs. This helped them to provide woman-centered care when returning to work.

Talking about it a lot. About the cases that you have experienced. Talking about it with colleagues from work or I have contacted [name of colleague] about it, for example. In any case, talking about it very much, so I can be a little bit [calmer]; when I am home, I am home, and when I am in the delivery room, I am in the delivery room with the woman. (Midwife 11)

The emotional side of working as a midwife surfaced repeatedly in their narrations. However, besides effective coping strategies to remain grounded, midwives also needed a strong emotional basis to cope with distress, in particular, when they felt that women had experienced violence in their past.

Because there is so much pain, especially if emotional violence has been exerted over a longer period, then so much pain surfaces. A midwife has to be very tough to encounter that. (Midwife 6)

#### Intuition and sensing the woman

Midwives stressed intuition and sensing the woman as part of midwifery, which means being fully committed to the woman’s needs. In this context, midwives act empathically in relation to the woman’s needs and wishes, to prevent her from becoming distressed. In their own words, they “sensed” the woman:

I don’t think during midwifery but I sense what the woman needs. Or I detect [what is needed] before there might be a problem. And that is what characterizes me especially as a midwife. (Midwife 1)

In general, midwives tried to be open to subtle hints from the woman and her relationship with her partner to see how they could provide her with the best care. This openness and sensing of the woman supported understanding of her behavioral cues, such as panic or being tense during examination. Such cues were assessed to determine if women had experienced violence.

That makes it very difficult in our profession because often you have patients to whom you can connect only with great difficulty or when you think “Oh, she is strange.” Thus, this is the first impression. And when you think about it a bit more . there is somehow “Hm, I think there is something deep down and she does not want to tell me right now.” (Midwife 15)

This standard of caring for women conflicted with how they perceived the health system. Challenges with the health system were experienced as twofold. One challenge was with woman-centered care and adapting to the woman’s needs, as shown in the example below:

[.] I have met one or two woman who, yes, I am almost certain that something must have happened [to them]. [.] you noticed during vaginal examination that it was unbelievably painful for these women, they really shut down and it was really difficult to deal with it, especially as the student I was back then. And to feel that because of the hospital regulations you should vaginally examine them now but also thinking “I don’t think it is necessary now, now in this very moment.” (Midwife 8)

This example shows that midwives sensed when women needed a more relaxed, rather hands-off approach and often suspected violence in such cases. However, midwives felt that this “sensing” was in contrast to regulations regarding how to “do” labor and what was expected from them as midwives by the organization. The current health system was experienced as challenging for continuous care as it consisted of different, independent actors (e.g., medical doctors, midwives in private practice). Thus, relevant information was gathered by every actor in the health system, which was seen as difficult in the context of violence and providing optimal care.

Because it is all so multi-layered; there is the midwife in private practice who does postpartum care, then there is the doctor in private practice, then there is the hospital. And everyone takes his own medical history with the patient and there is nothing continuous, where the hospital gets any information from the doctor or the midwife or anyone. Therefore . it is difficult. (Midwife 8)

#### Protecting, guiding, and empowering

Being a protector was noticeable in reports of protecting women from harm in terms of OV but also when midwives discussed empowering women and fostering their self-determination and self-efficacy. Protecting women from harm or violation was associated with a certain amount of experience and self-confidence that would allow the midwife to stand up against others.

[.] in the delivery room, it [a violation of the woman] still might happen to me, if there was a doctor where something like that would happen. But now I would be better at giving him a piece of my mind, and I would be much more direct, and I would be able to shield the woman better. Back then, I was hardly able to look at myself in the mirror because I felt I had failed to protect the woman. (Midwife 8)

The example demonstrates the importance of actively protecting women in their care. It additionally shows the visible connection between OV and childbirth that leads midwives to intervene to protect women from harm. Similar interventions were detected in midwives’ efforts to empower women. As shown in this example, empowering women meant supporting them to express their needs and wishes:

[It does not have to be] like, “I need to lie down, and then the doctor comes in, and he has to examine me.” That this is not the case, that she can say, “Excuse me, I am in pain right now” or “I don’t want that right now, because it hurts me.” (Midwife 13)

Fostering self-determination and self-efficacy in women meant supporting women in stating what they want and do not want during examinations. It also meant respecting and not interfering with women’s wishes. An example of fostering self-determination and not interfering with the woman’s wishes meant to respect the wish not to be examined. These attitudes were particularly pronounced in cases of suspected abuse. In such cases, midwives adjusted their behavior, and if possible, only one midwife consistently cared for the woman.

This one woman [with a history of violence] was seldom examined and only by one midwife, the teaching midwife. Not by the doctor or anyone else. That was handled in a really restrictive manner. And she . was, I think, she was ok with that because she was able to build a bond of trust very quickly with the midwife. (Midwife 8)

In cases of (suspected) violence, midwives reduced the number of vaginal exams and tried to hand over as much control to the woman as possible by explaining every step of the examination or by agreeing on a signal in case the examination became too intrusive or unbearable.

Maybe she talks about it [violence], or I can ask her that I will examine her now to have a look at how far along the dilation is, where the head is. Then it can be that she tells me: I am scared of the examination. Or during the examination itself, I do not explicitly ask, [.] these women have the feeling of powerlessness, that they could not control what is happening to them. “I’ll hold your hand, and if you want me to stop, you just press my hand. And when it is ok, I will go a bit further.” Thereby she has the feeling that she can control the process. And these women tell me sometimes before but mostly afterward that they have bad memories. (Midwife 2)

#### Staying active in the context of DV

Asking about DV also meant being prepared for referral, actively initiating referral, and establishing a support network for women, if required. This meant that midwives planned which steps to take if there was a positive disclosure when they asked about DV. Midwives who had already started to ask about violence reported that they actively engaged with other professionals to ensure care for women affected by violence. Such active guidance was also described in the following example:

[I]f I have a suspicion [that this woman is experiencing DV], whom do I refer her to, what do I tell the woman? And it is most difficult if I only say to the woman, “you can call here or there.” [I] don’t know if she’ll do that. It’s easier to say, “can I have your phone number, I’ll call you [when I have arranged a meeting with a special institution/therapist]” and [.] then I’ll call her and say “let’s do it, let’s do this talk,” and she’ll be like “ok, yes.” So, getting a call is easier than just saying, “call them.” Because maybe you do that and the line is busy, and that’s that. [.] It has to be easy; it has to be automatic, I think. (Midwife 12)

Networking with other professionals and having a trusted referral network empowered midwives to ask about violence. Such a network was, at most times, actively constructed, particularly if midwives worked in private practice.

I am also in contact with the child protection service. [.] I have their folders and contact details, and I even referred a woman to them. Because I am not only involved in prevention but also in post-pregnancy care, and I do mother-child counseling, and I had a mother who had a problematic situation, and I told her to go there. (Midwife 12)

It became obvious that some midwives saw referral as a kind of passiveness, such as handing the patient over to others. This perception can be discussed as a collision with the midwife’s active role. Such collision was noticeable when they did not know what had happened to their patients after referral or were out of touch after the child was born. The midwives experienced this as being difficult, not knowing how the woman was cared for after referral and if they were well taken care of.

In any case, she was traumatized in some way. Unfortunately, I don’t know what happened next [after the referral], because we called someone from the psychology unit, where she was taken care of, up to a given point. (Midwife 3)

In this sense, they were also saying that “[.] what happened after [the referral], you, unfortunately, do not hear about it as a student” (Midwife 5). Some midwives stressed the hierarchical structure in which knowledge traveled, whereby some receive information, and others do not. This was overcome by gaining experience and confidence as a professional and gaining status as a midwife. The same midwife described that within the legal possibilities regarding confidentiality, she followed up on every patient.

I trace every case, no matter if it was a case where I suspected that something had happened [e.g., DV] or a difficult birth. I trace everything, and now I also have the competence to be able to say that I want to know what happened after [giving birth]. (Midwife 5)

## Discussion

This study sought to explore midwives’ perceptions of and experiences with VAW in midwifery using a qualitative approach. From our findings, we concluded that VAW, especially DV, is part of the protective and active role of midwives, despite their hesitation to discuss DV with women in their care. This protective role was particularly noticeable when there was a visible connection between VAW and pregnancy, childbirth, or puerperium. This conclusion was found to be a novel aspect, as such a connection has hardly been addressed in previous studies.

Protecting women and being empathic and sensitive toward their needs and wishes have also been discussed concerning the with-woman approach in midwifery (Bradfield, Hauck, Kelly, & Duggan, 2019; Fontein-Kuipers et al., 2018). This approach focuses on the relationship, collaboration, and trust between the woman and midwife. In this study, the with-woman approach was found when midwives referred to adjusting their behavior (e.g., reducing the number of vaginal exams) to the woman’s needs, and when they attempted to empower women by encouraging them to express their wishes. Midwives are “guided guide[s]” (Thelin et al., 2014, p. 115) and protectors of women (Levy, 2006). In light of our findings, we assume that such guides and protectors can best operate when they are aware of the connection between VAW and caring for women. However, this connection was addressed more with regard to OV than DV. We assume that owing to campaigns such as the Roses Revolution (2013) and the WHO’s (2018) recommendation of respectful maternity care, OV was particularly highlighted when referring to the active protection of women. This stood in contrast to the caution regarding other forms of VAW, such as DV or IPV. This caution was most noticeable because midwives discuss this issue with the women in their care, despite their suspicion of violence. When midwives did not initiate conversations about violence and its impact on pregnancy, childbirth, and puerperium, the active protection was notably reduced. Lowered self-efficacy can be noted when midwives stated that they did not know how to broach the topic. These restraints on midwives’ actions might also be related to a lack of control over a situation (Oelhafen & Cignacco, 2018), which could also be related to uncontrollability, helplessness, and passivity in DV situations. This reflects the fact that women affected by DV experience uncontrollability and helplessness (Engnes et al., 2012), which in turn is transferred to midwives.

Reduced active protection might also be connected to status and experience as a midwife. Some midwives stated that as students, they were not given information, or that their inexperience contributed to their inability to stand up to others and protect women. On this note, lack of training, knowledge, and skills regarding what to do when a woman has experienced DV might also lead to feelings of uncontrollability and, thus, reduced self-efficacy in midwives.

These considerations corroborate our theory that midwives’ active role as protectors is connected to the need to actively address VAW, particularly DV and IPV. In this sense, actively addressing and discussing VAW with affected women is consistent with midwives’ professional role. In Henriksen et al.’s (2017) study, midwives’ role meant having the sensitivity and proximity to discuss IPV with women in their care.

Having personal interest in the subject of IPV has facilitated midwives asking about violence (Henriksen et al., 2017). Our findings support this assumption, as we found that midwives’ role as protectors and guides stimulates the broaching of this topic. The hidden or visible connection to different forms of VAW might encourage midwives to act according to their professional role. To include the subject of VAW as an aspect of midwifery, we need to address both the professional role of midwives and their training, which was visibly lacking in our sample. Other studies have found that midwives are hesitant when it comes to actively addressing DV (Eustace et al., 2016; Mauri et al., 2015), which was often due to lack of training or support (Finnbogadóttir & Dykes, 2012; Pitter, 2016) and the lack of support structures and resources for midwives (Finnbogadóttir & Dykes, 2012; Henriksen et al., 2017). Our findings corroborate these studies. As also illustrated by our core category, “protecting but walking on eggshells,” we observed a constant tension between being a protector and including DV routine inquiry as an aspect of midwifery. Thus, on one hand, midwives adopt the with-woman approach and offer protection, while on the other hand, they are cautious about broaching the topic of DV. This tension and ambiguity have the power to silence women in DV contexts (Towns & Adams, 2016), while midwives’ ambivalence toward an active discussion of DV further contributes to silencing of this subject in midwifery.

### Limitations

This study has a number of limitations. The small sample size meant that the findings must be interpreted with care. Although we achieved data saturation during analysis, our sample included a small group of midwives in a particular part of Austria and the sample was demographically homogenously, except for age. Recruitment of participants was difficult, contributing to a sample of only 15 midwives. These difficulties might also suggest that the subject of violence has not yet been fully acknowledged in midwifery. While we invited all midwives in this region to participate, we were most successful in recruiting participants face-to-face at their annual meeting. Thus, participants were already those who demonstrated active engagement as midwives by attending the annual plenary meeting. Nevertheless, the midwives agreed with the derived storyline when they were informed about the findings at one of the following annual regional meetings. We presented our findings at an annual regional plenary meeting to encourage midwives to engage in our endeavor to fight VAW (Thomas, 2017) and in future projects on including DV in midwifery.

We found that our study findings resonated with other research in Western countries. An Italian study demonstrated that midwives felt unprepared to screen for IPV (Mauri et al., 2015); an Australian study also reported that midwives felt unprepared, lacked support, and were concerned regarding disclosure of IPV (Eustace et al., 2016); an Irish study revealed that midwives lacked skills regarding how to initiate a conversation on IPV (Carroll et al., 2018); a Swedish study found that midwives feared they could fail the mother and neonate if the mother had experienced IPV (Finnbogadóttir & Dykes, 2012). Thus, our findings are not unique to the Austrian context. Although VAW or DV training was reported during the interview, we did not focus on whether midwives themselves were VAW survivors, or how this might affect their actions and attitudes toward VAW. This aspect will be included in our follow-up project.

Initially working in a hospital or private practice, or working in a rural or urban environment appeared to make a difference in terms of the perception of VAW and whether or not to screen for it. However, it became clear that this did not influence perception or actions. Diversity in terms of age and experience did not influence what topics were discussed in the interviews; however, such diversity proved important when it came to status as a midwife and possibilities for protection.

## Conclusions

In this study, we found that the active protecting role of midwives was associated with dealing with VAW in the care of women. Caution when addressing DV was noticeable, but can be explained by midwives’ ambivalence concerning their role as protector and actively intervening if harm occurs. To initiate an active intervention, the immediate impact of VAW had to be visibly associated with pregnancy, labor, or puerperium. Naturally, DV has an impact on pregnancy, labor, and puerperium; however, DV remains invisible compared to incidents of OV occurring on-site, child removal, or stillborn neonates. Addressing this “invisibility” and midwives’ active engagement in reducing VAW in midwifery education and advanced training of midwives is expected to achieve sustainable inclusion of VAW routine inquiry in midwifery. A systematic review of the screening for IPV concluded that insufficient evidence is available regarding the screening of every woman for IPV in health care settings (O’Doherty et al., 2015). Similarly, a systematic review on the safety and effectiveness of interventions reported inconsistent empirical findings and called for more high-quality studies in the field of IPV, pregnancy, and interventions (Jahanfar et al., 2014). Currently, recommendations state that professionals should be properly trained to make routine inquiries during maternal care (WHO, 2016). Based on our findings, we have the following recommendations for policy and practice:

- Make DV visible in midwifery: include evidence-based information regarding DV and VAW in the education and advanced training of midwives. This information should highlight the effects of DV and VAW throughout pregnancy and during childbirth (Leeners et al., 2016; LoGiudice, 2017; Montgomery et al., 2015), on birth outcomes (Alhusen et al., 2015; Meuleners et al., 2011), and in accessing antenatal care (Jamieson, 2018; Musa et al., 2019).
- Include the active participation of midwives to reduce VAW: training in routine inquiries should include the midwife’s professional role in actively protecting women from harm. Midwives play a crucial role in identifying and supporting women affected by VAW. Strengthening midwives in this role part will help to combat VAW and ultimately reduce VAW.
- Strengthen the protective role of midwives by emphasizing these aspects during interventions. Asking about and responding to VAW creates a woman-centered approach in midwifery. Routine inquiries should be perceived as a form of protection, not merely as a means of identifying women experiencing VAW.
